# Sex- and Age-Dependent Changes in the Adiponectin/Leptin Ratio in Experimental Diet-Induced Obesity in Mice

**DOI:** 10.3390/nu15010073

**Published:** 2022-12-23

**Authors:** Sara Becerril, Amaia Rodríguez, Victoria Catalán, Beatriz Ramírez, Amaia Mentxaka, Gabriela Neira, Javier Gómez-Ambrosi, Gema Frühbeck

**Affiliations:** 1 Metabolic Research Laboratory, Clínica Universidad de Navarra, 31008 Pamplona, Spain; 2 CIBER Fisiopatología de la Obesidad y Nutrición (CIBEROBN), Instituto de Salud Carlos III, 28029 Madrid, Spain; 3 Obesity and Adipobiology Group, Instituto de Investigación Sanitaria de Navarra (IdiSNA), 31008 Pamplona, Spain; 4 Department of Endocrinology & Nutrition, Clínica Universidad de Navarra, 31008 Pamplona, Spain

**Keywords:** leptin, adiponectin, adiponectin/leptin ratio, female, obesity

## Abstract

Biological sex and aging impact obesity development and type 2 diabetes, changing the secretion of leptin and adiponectin. The balance between these factors has been propounded as a reliable biomarker of adipose tissue dysfunction. Our proposal was to study sexual differences and aging on the adiponectin/leptin (Adpn/Lep) ratio in order to acquire a broader view of the impact of consuming an high-fat diet (HFD) on energy metabolism according to sex and age. Male and female C57BL/6J mice were fed a normal chow diet or an HFD for 12 or 32 weeks (*n* = 7–10 per group) and evolution of body weight, food intake and metabolic profile were registered. The HFD triggered an increase in body weight (*p* < 0.001), body weight gain (*p* < 0.01) and adiposity index (*p* < 0.01) in both sexes at 32 weeks of age, but female mice fed the HFD exhibited these changes to a significantly lower extent than males. Aged female mice showed an increase (*p* < 0.01) in the Adpn/Lep ratio, which was negatively correlated with body weight gain, changes in different fat depots and insulin resistance. Females were more metabolically protected from obesity development and its related comorbidities than males regardless of age, making the Adpn/Lep ratio a relevant factor for body composition and glucose metabolism.

## 1. Introduction

Type 2 diabetes (T2D) prevalence is growing worldwide concomitantly with obesity, emerging as a global international public health issue [1,2,3]. Obesity is a complex, multifactorial and preventable disease; the most relevant factors that influence energy homeostasis are increased food intake, decreased energy expenditure, fat accumulation and nutrient absorption [4]. Dietary fat induces obesity and its related comorbidities in both humans and mice [5]. The distribution of adipose tissue (AT) differs substantially depending on biological sex, thereby exerting an influence on the development and pathogenesis of these metabolic diseases. Epidemiological studies have reported relevant sex differences in insulin resistance incidence, and T2D has a much higher prevalence in men than in women [6,7].

The C57BL/6 J inbred strain of laboratory mouse is considered a particularly good animal model for mimicking human metabolic disorders since these rodents develop obesity, hyperinsulinemia, hyperglycemia and hypertension when allowed ad libitum access to an high-fat diet (HFD) [8]. Male mice resemble humans in that they are more susceptible to diet-induced obesity (DIO) and exhibit lower insulin sensitivity than female mice, whereas females are more likely to be protected from the development of these pathologies [9,10,11]. However, the underlying mechanisms that drive this sexual dimorphism in metabolism have not yet been elucidated.

Obesity is also associated with a state of chronic low-grade inflammation with immune cell infiltration in AT. Immunophenotype alterations derived from aging constitute an important contributory factor to the pathogenesis of the metabolic disorders of obesity, most notably insulin resistance, steatosis, and generalized dyslipidemia. Healthy or young AT exhibits an adequate metabolic response, with the infiltrated immune cells being responsible for maintaining homeostasis through the secretion of anti-inflammatory cytokines. The proinflammatory immune cells present in obese or aged AT promote its dysfunction, contributing to the development of insulin resistance [12].

Both biological sex and aging change the secretion of hormones and cytokines related to energy metabolism, including leptin, adiponectin or insulin, contributing to the development of obesity-related diseases. In this sense, adiponectin and leptin have a shared origin, as they are mainly secreted by adipocytes in white AT (WAT), but their activities are quite different. Adiponectin possesses insulin-sensitizing, anti-inflammatory and antiatherogenic properties and its plasma levels are negatively associated with percent body fat and insulin. Leptin is principally responsible for the regulation of feeding behavior and energy balance as it is involved in physiological functions that include hematopoiesis, angiogenesis and reproduction, among others; it is produced in proportion to body fat stores [13,14,15]. The balance between these AT- derived factors, the adiponectin/leptin (Adpn/Lep) ratio, has been proposed as a reliable biomarker of the AT dysfunction and inflammation, corresponding more closely with insulin resistance than adiponectin and leptin separately and decreasing with the augmented number of metabolic risk factors [16,17]. Both sex and age differences impact biological processes, but it is not yet known the extent to which the aging-related increase in AT and the simultaneous effect of sex in a model of DIO promotes metabolic homeostasis alterations. In this sense, although the association between aging and increased prevalence of T2D has long been recognized [18,19], the potential contribution of sex-dependent mechanisms to aging-associated insulin resistance remains unexplored. We hypothesized that females are more likely to be protected from developing obesity and its related comorbidities with aging than males, with the Adpn/Lep ratio constituting a significant factor as well as a relevant marker of metabolism. To that end, we analyzed the effect of aging and sexual differences on the Adpn/Lep ratio in order to gain a broader view of AT function according to sex and age. We have compared cohorts of male and female C57BL/6J mice under standard chow diet and HFD conditions for 12 or 32 weeks to observe the effect of both variables together with the impact of an HFD on energy homeostasis.

## 2. Materials and Methods

### 2.1. Experimental Animals

Male and female C57BL/6J mice pups were weaned between 21 and 23 days of age, housed (2–3 animals per cage) and maintained at room temperature (22 ± 2 °C), with a relative humidity (50 ± 10%), artificial light–dark cycle (lights on from 8:00 am) and under pathogen-free conditions. The mature adult mice ranged in age from 3 to 6 months (12 to 24 weeks), so they were not yet affected by senescence. After six months, mice are considered middle aged and some age-related changes may be present [20]. For this purpose, a cohort of male and female mice aged 12 weeks (“mature”) and 32 weeks (“aged”) were selected. Animals had free access to tap water and were fed *ad libitum* either a normal diet (ND) (2014S Teklad Global 14% Protein Rodent Maintenance Diet, Harlan, Barcelona, Spain) or an HFD (D12451 Research Diets, Inc., New Brunswick, NJ, USA, 45% kcal fat) (*n* = 7–10 per group). Evolution of body weight (BW) and food intake were registered twice weekly during the experimental period (12 and 32 weeks). Calculations were made based on the average food intake of each cage, standardized to the individual body weights. The food efficiency ratio (FER) was calculated as a ratio of BW (grams) gained per week divided by total energy (kilocalories) consumed over this period. Rectal thermoprobe was used to measure body core temperature in mice (YSI 4600 Series Precision Thermometers, YSI Temperature, Dayton, OH, USA).

### 2.2. Blood and Tissue Collection

12- and 32-week-old mice were sacrificed by CO_2_ euthanasia in a fasting condition. Heart puncture was used for blood collection, and sera were obtained by cold centrifugation (4 °C) at 700× *g* for 15 min and stored at −20 °C. Gonadal, subcutaneous, perirenal and brown fat depots were carefully excised. Tissue and plasma samples were immediately stored at −80 °C for subsequent assays. Total body fat was measured as the sum of gonadal, perirenal and subcutaneous fat pad weights. The adiposity index was determined as (total body fat/final BW) × 100 (Supplementary Figure S1). All experimental procedures conformed to the European Guidelines for the Care and Use of Laboratory Animals (directive 2010/63/EU). The study was approved by the Ethical Committee for Animal Experimentation of the University of Navarra (042/03, 041/08).

### 2.3. Blood Measurements

Serum concentrations of free fatty acids (FFA) (Wako Chemicals, GmbH, Neuss, Germany) were determined by enzymatic methods using commercially available kits [21]. Insulin, adiponectin and leptin were determined by ELISA (Crystal Chem, Inc., Chicago, IL, USA) as formerly indicated [14,22]. Intra- and inter-assay coefficients of variation for measurements were 4.5% and 5.4%, 3.5% and 6.3%, and 5.6% and 7.2%, respectively. To estimate insulin resistance, the Homeostatic Model Assessment (HOMA) index was calculated as fasting insulin concentration (μU/mL) × fasting glucose concentration (mmol/L)/22.5. The quantitative insulin sensitivity check index (QUICKI), an indirect measure of insulin sensitivity, was determined as follows: 1/(log(fasting insulin μU/mL) + log(fasting glucose mg/dL)). The adipose tissue insulin resistance index (Adipo-IR), a surrogate measure of adipocyte dysfunction, was calculated as fasting FFA (mmol/L) × fasting insulin (pmol/L).

### 2.4. Oral Glucose Tolerance Tests

Blood glucose was measured in conscious male and female mice before (baseline) and 15, 30, 60 and 120 min after the administration of a D-glucose solution (2 g/kg of BW) by oral gavage after 12 h of fasting. Glucose was measured by glucometer (Ascensia Elite, Bayer, Barcelona, Spain) from whole blood samples collected by puncturing the tail vein. The area under the curve (AUC) was calculated from the oral glucose tolerance test (OGTT) curve by the trapezoidal method.

### 2.5. Intraperitoneal Insulin Tolerance Test

Male mice were fasted for 5 h before intraperitoneal injection with insulin (0.5 units/kg). Glucose concentrations were determined by glucometer (Ascensia Elite) before and 15, 30, 60 and 120 min after the insulin administration from whole blood samples collected by puncturing the tail vein. The area under the curve (AUC) was calculated from the intraperitoneal insulin tolerance test (IPITT) curve by the trapezoidal method.

### 2.6. Statistical Analysis

Data are mean ± Standard Error of the Mean (SEM) of 7–10 animals per group. Normality was assessed by the Kolmogorov–Smirnov test. For each group, the control is the mice of the same sex, fed a ND. Differences between groups were tested by two-way ANOVA and, in case of interaction between factors (sex and diet), one-way ANOVA followed by Bonferroni’s post hoc tests were applied. Pearson’s correlation coefficient (*r*) was used to analyze the association between variables. Statistics were calculated using the SPSS/Windows version 15.0 software (SPSS, Inc., Chicago, IL, USA) and the figures were created using GraphPad Prism version 8.3 (GraphPad Software, Inc., San Diego, CA, USA). A *p* value < 0.05 was considered statistically significant.

## 3. Results

### 3.1. Effect of Sexual Differences and Diet on Energy Homeostasis

**Figure 1:**
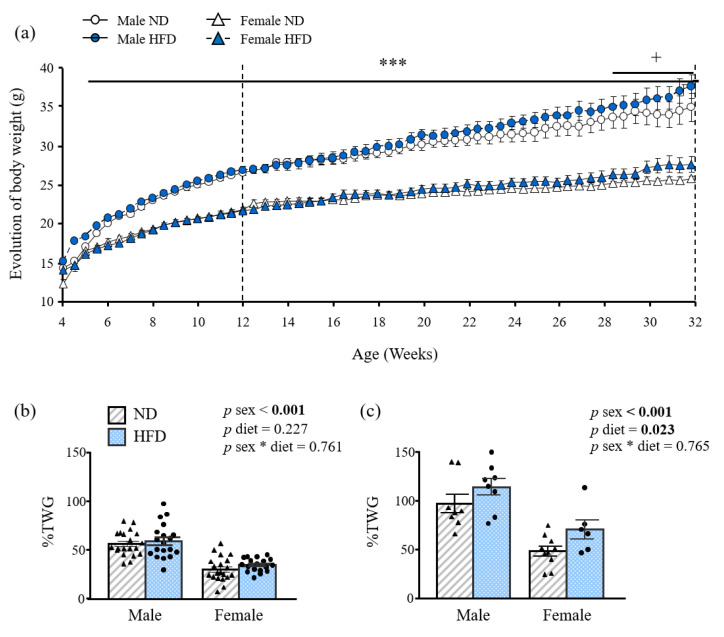
Growth curves of 4–32 week-old-male and female mice fed a normal (ND) or an high- fat diet (HFD) (**a**). Bar graphs show the % total weight gain (TWG) of (**b**) 12- and (**c**) 32- week-old mice (*n* = 10–20). Data are the mean ± SEM. Statistical differences were analyzed by two-way ANOVA. * interaction between both factors (sex and diet); *** *p* < 0.001 effect of sex; + *p* < 0.05 effect of diet. *p* values lower than 0.05 are highlighted with bold text.

In order to determine if the time course of DIO is sex divergent, BW and food intake of both males and females was measured over 32 weeks. Following 12 weeks of HFD, the BW and total BW gain (%TWG) of female mice was significantly lower (*p* < 0.001) than that of male mice (Table 1), although there were no differences in %TWG compared to ND (Figure 1a,b). After 32 weeks of HFD intake, BW was increased by 19 g in males and 13 g in females, corresponding to a TWG of 115% in males and a 71% in female mice. HFD-fed female mice exhibited these changes to a significantly lower extent (*p* < 0.001) than males (Figure 1c) in spite of showing increased relative food intake (*p* < 0.001), expressed as kcal/100 g of BW (Table 1). Interestingly, 12- and 32-week-old HFD female mice showed decreased (*p* < 0.01 and *p* < 0.05, respectively) rectal temperature as compared to male mice. However, FER of female mice, a measure of the animal’s efficiency to convert feed into body mass, was significantly reduced (*p* < 0.001) as compared with that of male mice at both 12 and 32-weeks of age.

Both 12- and 32-week-old male mice fed an HFD showed significantly increased gonadal, subcutaneous, perirenal and total fat pads (*p* < 0.001 all) compared to their respective ND controls. Moreover, HFD increased fat mass to a greater extent in 12- and 32-week-old males than females (Table 1). In this sense, the lower TWG observed in HFD female mice was related to a significant reduction in gonadal, subcutaneous and total fat depots compared to male mice (*p* < 0.001 all). No differences in brown adipose tissue (BAT) were observed among young groups, whereas a significant effect of sex was observed at 32 weeks of age (*p* < 0.001) (Table 1).

As expected, 32-week-old male mice fed an HFD showed significantly augmented leptin levels compared to males fed a ND (*p* < 0.001) and HFD-fed females (*p* < 0.001). A positive correlation with the adiposity index was also detected (*r* = 0.708; *p* < 0.001). The 12-week-old ND female mice exhibited increased Adpn levels (*p* < 0.05) as compared to males, but these differences disappeared with age.

### 3.2. Sex and Age-Dependent Differences in Glucose Metabolism

In fasting conditions, 12-week-old male and female mice exhibited no changes in glycemia by diet, with the basal glucose levels in females significantly lower (*p* < 0.05) than in males (Table 2). At 32 weeks of age, however, HFD increased basal glucose levels in both sexes (*p* < 0.001), with females exhibiting significantly lower glucose levels (*p* < 0.01) than males (Table 2).

**Figure 2:**
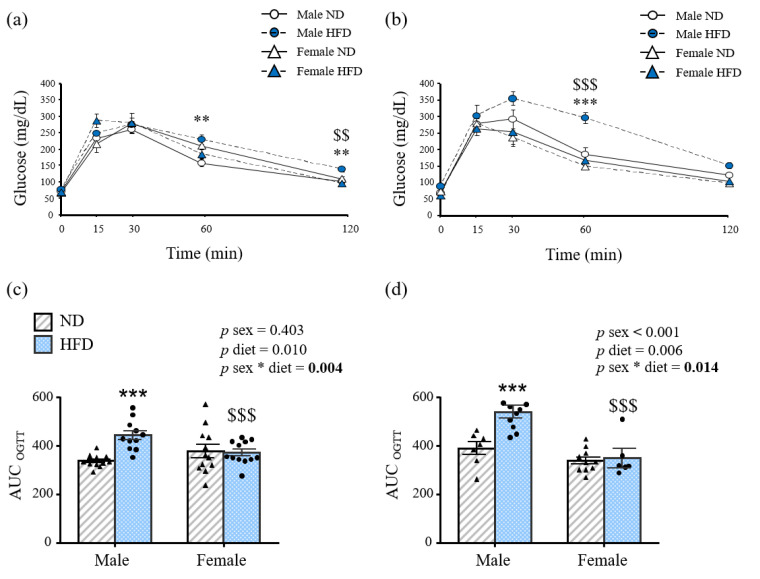
Oral glucose tolerance test of (**a**) 12- and (**b**) 32-week-old mice and area under the curve of (**c**) 12- and (**d**) 32-week-old experimental animals. Data are presented as the mean ± SEM of 6–12 animals per group. Statistical differences were analyzed by two-way ANOVA or one-way ANOVA followed by Bonferroni post hoc test if an interaction was detected. * interaction between both factors (sex and diet); ** *p* < 0.01; *** *p* < 0.001 *vs*. male mice fed a ND; $ *p* < 0.05; $$ *p* < 0.01; $$$ *p* < 0.001 *vs*. female mice fed the same diet. AUC, area under the curve, HFD, high-fat diet; ND, normal diet; OGTT, oral glucose tolerance test. *p* values lower than 0.05 are highlighted with bold text.

Glucose control, examined by OGTT, was also affected in a sexually dimorphic manner. Male mice on HFD showed worsened glycemia at the end of the OGTT compared to ND-male and the HFD-female mice at both ages (Figure 2a,b). Glycemia 60 and 120 min after the glucose load was significantly higher in 12-week-old males fed an HFD than ND-fed ones (*p* < 0.01), whereas these differences were not observed in female mice in the same conditions. Furthermore, male mice displayed significantly higher glycemia than female counterparts 120 min after the glucose load (*p* < 0.01) (Figure 2a). These sex differences were also observed in 32-week-old males fed an HFD compared to females from 30 min to the end of the experiment (all *p* < 0.001) (Figure 2b).

Data reveal a statistically significant interaction between sex and diet for the AUC at both ages (*p* < 0.01). In this sense, sex did not affect the AUC after the OGTT in mice fed a ND (Figure 2b,c). The glucose AUC was significantly higher in HFD male compared to ND male mice at both ages (*p* < 0.001 for both), whereas HFD female mice showed significantly lower AUC than males (*p* < 0.001) (Figure 2c,d), highlighting an impaired glucose tolerance in males. Furthermore, 12-week-old HFD male mice exhibited decreased insulin sensitivity as evidenced by the increased concentrations of insulin (*p* < 0.01), HOMA and adipo-IR indexes (*p* < 0.05) (Table 2). These differences, together with a significant decrease (*p* < 0.001) in the QUICKI index were also observed at 32 weeks of age (Table 2).

### 3.3. Sex-Specific Effect of HFD on Adiponectin/Leptin Ratio

**Figure 3:**
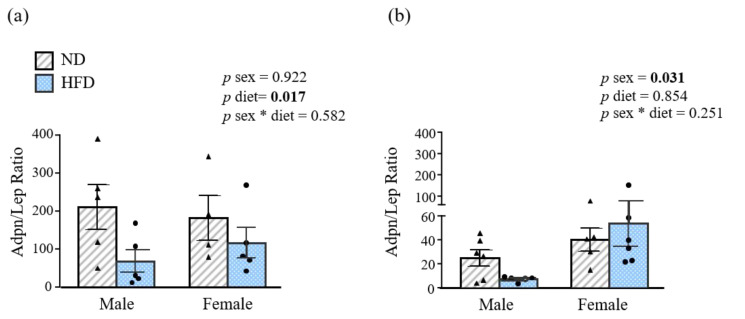
Adiponectin/leptin (Adpn/Lep) ratio of (**a**) 12- and (**b**) 32-week-old mice. Data are presented as the mean ± SEM of 6–8 animals per group. Statistical differences were analyzed by two-way ANOVA. * interaction between both factors (sex and diet); Adpn, adiponectin; HFD, high-fat diet; Lep, leptin; ND, normal diet; *p* values lower than 0.05 are highlighted with bold text.

The Adpn/Lep ratio, a functional biomarker of dysfunctional AT, significantly decreased (*p* < 0.05) with the HFD at 12 weeks of age in both sexes. As expected, 32-week-old female mice exhibited a significantly increased (*p* < 0.05) Adpn/Lep ratio compared to males (Figure 3a,b).

**Figure 4:**
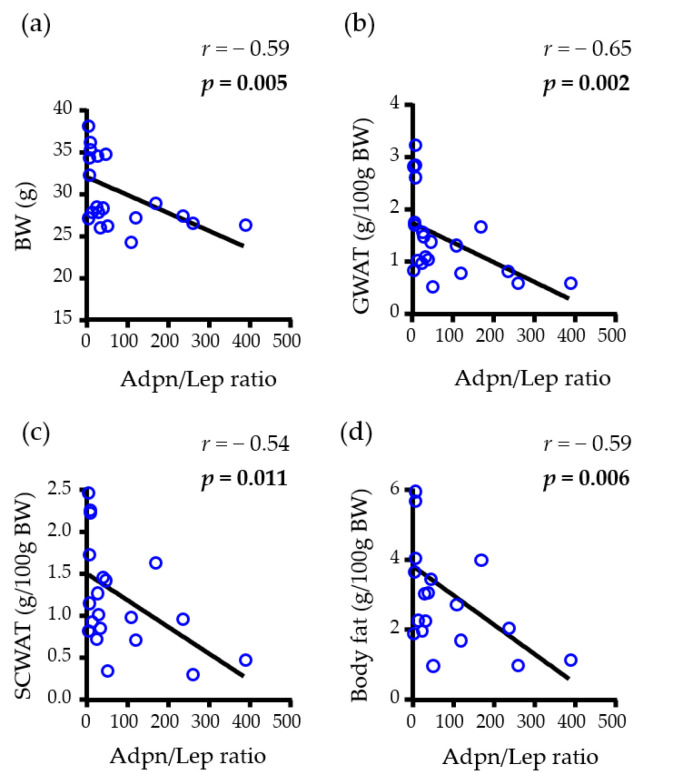
Univariate analysis of the correlations between the adiponectin/leptin (Adpn/Lep) ratio with (**a**) body weight (BW), (**b**) gonadal (GWAT), (**c**) subcutaneous (SCWAT) and (**d**) whole-body fat content of male animal. Adpn/Lep ratio, adiponectin/leptin ratio; BW, body weight; *r*, Pearson correlation coefficient; *p*, significance value. *p* values lower than 0.05 are highlighted with bold text.

Focusing on male mice, a negative correlation of the Adpn/Lep ratio with BW (*r* = −0.59, *p* = 0.005) as well as with different fat depots, including GWAT (*r* = −0.65, *p* = 0.002), SCWAT (*r* = −0.54, *p* = 0.011) and total WAT (*r* = −0.59, *p* = 0.006) (Figure 4) was found. No correlation with basal glucose was observed.

**Figure 5:**
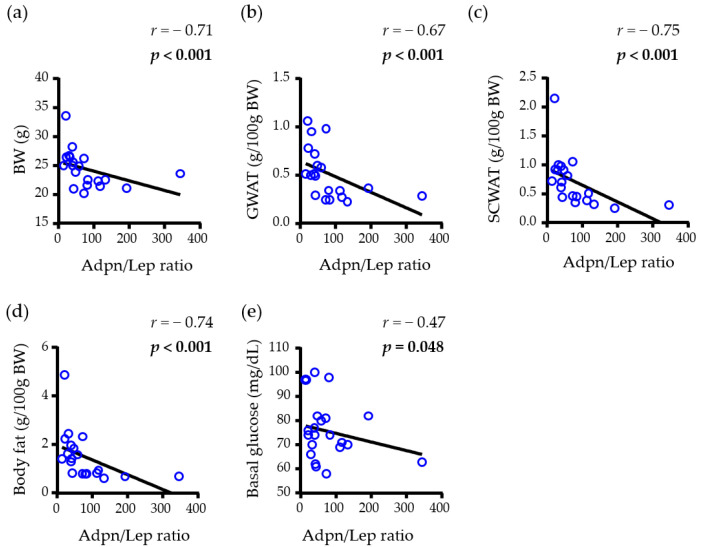
Univariate analysis of the correlations between the adiponectin/leptin (Adpn/Lep) ratio with (**a**) body weight (BW), (**b**) gonadal (GWAT), (**c**) subcutaneous (SCWAT) and (**d**) whole-body fat content of female animal, together with the basal glucose (**e**). Adpn/Lep ratio, adiponectin/leptin ratio; BW, body weight; *r*, Pearson correlation coefficient; *p*, significance value. *p* values lower than 0.05 are highlighted with bold text.

Regarding female mice, a negative correlation between the Adpn/Lep ratio and BW (*r* = −0.71, *p* < 0.001), GWAT (*r* = −0.67 *p* < 0.001), SCWAT (*r* = −0.75, *p* < 0.001) and total WAT (*r* = −0.74, *p* < 0.001) was also observed. To further reinforce the accuracy of the Adpn/Lep ratio as an important marker of metabolic improvement, the correlation between the Adpn/Lep ratio and basal glucose was also analyzed, and a negative correlation between them was identified (*r* = −0.44, *p* = 0.048) (Figure 5).

## 4. Discussion

Despite the well-known differences in metabolic physiological systems, diseases and treatment outcomes among sex, the models used in the study of obesity are limited by the underrepresentation of females [23,24,25], as the presence of both sexes in animal as well as in cell-based studies has been recently considered [26]. In this context, it is necessary to examine the sex-specific characteristics of glucose metabolism as they are clearly involved in regulatory mechanisms in clinical pathologies including metabolic syndrome, obesity and T2D [27,28]. Obesity superimposed on aging drastically alters the inflammatory status, promoting the development of metabolic diseases, such as T2D, metabolic syndrome and cardiovascular disease [29]. However, to our knowledge, few publications delve into the simultaneous metabolic effect of sex and age on the response to an HFD. Since the strength of the reliability of the Adpn/Lep ratio as a biomarker of AT dysfunction has been previously described [30], the current study was focused on the age and sex-dependent effects of DIO on changes in the Adpn/Lep ratio, as well as in body composition and glucose metabolism.

Globally, many of the main findings that were related to the effect of HFD were in accordance with those previously published [31,32,33]. HFD and aging have a wide-ranging impact on metabolic variables, with a greater effect on male mice than female. Young HFD female mice showed a decreased body fat mass compared with females fed a ND despite their hyperphagia, which was probably due to their decreased FER. On the contrary, HFD induces an increased adiposity index in male mice compared with female ones, being male mice more prone to metabolic disorders associated with the HFD, even in the absence of overeating and their increased energy expenditure. The observed lower weight gain in females when presented with the metabolic challenge is in line with previous studies and was probably due to estrogens, which protect against increased obesity through their induced anorectic effects [34], as well as to lipolytic differences [35,36,37].

Sex- and age-dependent changes in plasma concentrations of adipokines and hormones related to glucose metabolism were also detected. The 12-week-old HFD female mice exhibited increased insulin sensitivity compared with males, as suggested by the decreased concentrations of plasma glucose and insulin as well as HOMA and adiposity indexes according to previous studies in both humans [38] and rodents [39]. Plasma adiponectin levels, widely described as an insulin sensitizing factor [40], were also increased in female mice. In this regard, aged HFD female rats showed improved glucose tolerance, suggesting a healthier serum profile of insulin sensitivity, which could be related with the increase in the AT insulin resistance index as well as in the Adpn/Lep ratio. We also observed that this biomarker correlated with body composition and insulin resistance better than adiponectin or leptin alone. However, these correlations were not observed in young females.

The combination of age and HFD significantly worsened the glucose tolerance of male mice, with aged male mice who were fed an HFD exhibiting hyperglycemia and glucose intolerance, thereby confirming their deleterious effects. The significant increase in serum adiponectin levels shown in aged male mice is interesting, contrasting with their impaired insulin sensitivity. Controversy related to the effect of an HFD on serum adiponectin levels exists [41,42], and a paradoxical increased incidence of death with higher adiponectin levels has been also reported [43]. The increased serum adiponectin levels observed in aged HFD male mice could be a compensatory mechanism to prevent insulin resistance, which was not sufficient to avoid the deterioration of systemic insulin sensitivity. The notion that females have improved insulin sensitivity is reinforced by the absence of differences as their age influences their glucose metabolism parameters. Sexual dimorphism with regard to insulin sensitivity has been well documented, and both clinical and animal studies exhibit a strong correlation between estrogen depletion, insulin resistance and dysregulation of metabolic homeostasis [44,45,46]. In this regard, estrogen may confer a protective role in the maintenance of insulin sensitivity in females [47] since this sex hormone is known to be a relevant modulator of glucose homeostasis, insulin signaling, energy balance and body composition [48]. In contrast to the preferential effect of estrogen on females, testosterone plays a key role in body composition, as well as in glucose and lipid metabolism, exerting an inhibitory effect on insulin sensitivity in males [49,50,51]. In line with these results, the differences in TWG observed between young males and females may also be associated with the effect of sex hormones, since testosterone favors muscle mass accretion [52], and differences disappear with age where testosterone concentrations are lower. A limitation of the present study is that adipocytes from rodent WAT were not isolated and cultured in order to study the hormone production related to energy homeostasis, and further investigations analyzing both the hormones and the mechanisms related to the improvement of the adipose tissue function in female mice are warranted.

**Figure 6:**
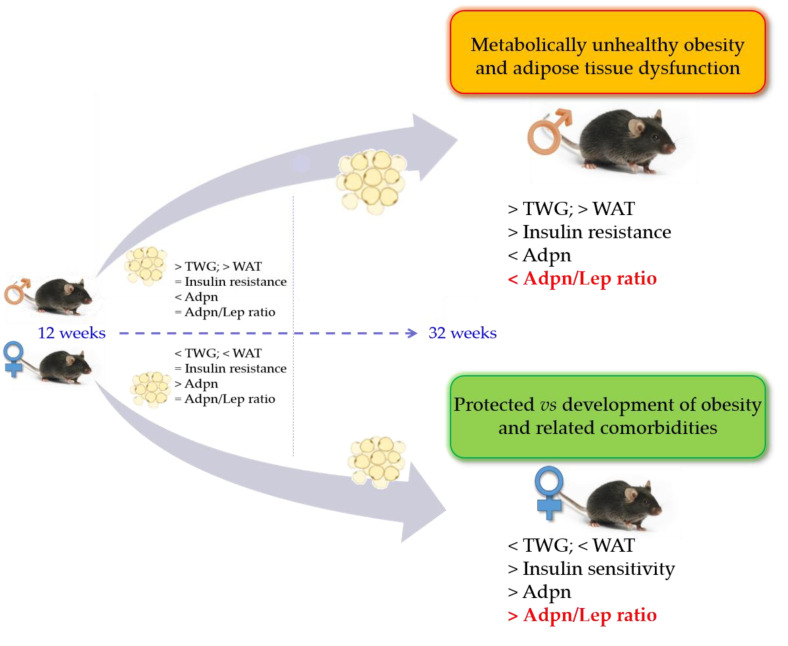
Impact of aging and sex in Adpn/Lep ratio on HFD-induced metabolic effects. Adpn, adiponectin; Adpn/Lep ratio, adiponectin/leptin ratio; TWG, total weight gain; WAT, white adipose tissue.

In summary, body composition and glucose metabolism are distinctly regulated in females and males and, together with age, influence the predisposition to the development of obesity and its associated comorbidities. In this sense, females are more metabolically protected against obesity and its associated metabolic disorders than males regardless of age, indicating that the Adpn/Lep ratio is a relevant biomarker of body composition and glucose metabolism (Figure 6). This study provides evidence that long-term HFD induces metabolic alterations during aging in a sex-dependent manner, meaning that sex is a critical factor to be considered in dietary strategies used to mitigate T2D development. Changes in body composition related to sex, age and diet of wild type animals might provide a reference standard to evaluate the effects of dietary factors, genetic manipulation or pharmacological treatments, constituting critical parameters for the potential application against metabolic diseases in humans.

## Figures and Tables

**Table 1:** Effect of sex and diet on the body weight, food intake, body composition and homeostasis in 12- and 32-week-old mice.

	12 WEEKS						
	Males		Females		ANOVA 12 weeks		
	ND	HFD	ND	HFD	*Sex*	*Diet*	Sex*Diet
BW(g)	26.1 ± 0.3	26.4 ± 0.6	21.6 ± 0.3	21.5 ± 0.2	**<0.001**	0.834	0.618
Relative FI (kcal/100 g BW)	42.8 ± 2.4	49.3 ± 1.4	54.5 ± 1.5	56.6 ± 1.3	**<0.001**	**0.023**	**0.246**
Rectal temp (°C)	36.7 ± 0.1	37.5 ± 0.2 ^b^	36.7 ± 0.1	36.8 ± 0.1 ^e^	0.010	0.001	**0.010**
FER	0.15 ± 0.01	0.17 ± 0.01	0.09 ± 0.01	0.14 ± 0.01	**<0.001**	**<0.001**	0.091
BAT (g/100 g BW)	0.31 ± 0.05	0.32 ± 0.02	0.30 ± 0.01	0.30 ± 0.01	0.309	0.691	0.587
GWAT (g/100 g BW)	0.65 ± 0.03	1.04 ± 0.09 ^c^	0.40 ± 0.03 ^e^	0.30 ± 0.03 ^f^	<0.001	0.010	**<0.001**
SCWAT (g/100 g BW)	0.56 ± 0.07	0.88 ± 0.09 ^c^	0.49 ± 0.06	0.45 ± 0.02 ^f^	0.001	0.023	**0.004**
PRWAT (g/100 g BW)	0.15 ± 0.02	0.33 ± 0.05 ^c^	0.13 ± 0.01	0.12 ± 0.02 ^f^	<0.001	0.003	**0.002**
Adiposity index (g/100 g BW)	1.35 ± 0.11	2.25 ± 0.23 ^c^	1.02 ± 0.11	0.87 ± 0.07 ^f^	<0.001	0.013	**<0.001**
Leptin (ng/mL)	0.60 ± 0.48	0.91 ± 0.29	0.24 ± 0.06	0.28 ± 0.05	0.132	0.565	0.680
Adpn (μg/mL)	23.1 ± 3.4	22.4 ± 1.4	34.5 ± 4.4	22.8 ± 1.9	**0.049**	**0.042**	0.065

	**32 WEEKS**						
	**Males**		**Females**		**ANOVA 32 weeks**		
	**ND**	**HFD**	**ND**	**HFD**	***Sex***	***Diet***	Sex*Diet
BW(g)	32.9 ± 1.2	36.9 ± 1.5	25.3 ± 0.3	27.2 ± 1.0	**<0.001**	**0.016**	0.547
Relative FI (kcal/100 g BW)	33.3 ± 1.5	34.9 ± 1.4	53.60 ± 1.49	47.06 ± 2.03	**<0.001**	**0.046**	0.104
Rectal temp (°C)	36.6 ± 0.1	37.5 ± 0.2 ^b^	37.1 ± 0.1 ^d^	36.8 ± 0.1 ^d^	0.349	0.046	**<0.001**
FER	0.05 ± 0.00	0.08 ± 0.01	0.01 ± 0.00	0.04 ± 0.01	**<0.001**	**0.011**	0.933
BAT (g/100 g BW)	0.44 ± 0.03	0.48 ± 0.04	0.34 ± 0.02	0.36 ± 0.02	**<0.001**	0.325	0.795
GWAT (g/100 g BW)	1.57 ± 0.15	2.31 ± 0.19 ^c^	0.68 ± 0.06 ^f^	0.81 ± 0.09 ^f^	<0.001	0.004	**0.038**
SCWAT (g/100 g BW)	1.26 ± 0.13	1.94 ± 0.17	0.85 ± 0.06	1.02 ± 0.15	**<0.001**	**0.004**	0.070
PRWAT (g/100 g BW)	0.50 ± 0.06	0.73 ± 0.05	0.27 ± 0.02	0.50 ± 0.14	**<0.001**	**0.007**	0.772
Adiposity index (g/100 g BW)	3.37 ± 0.34	4.85 ± 0.40	1.80 ± 0.13	2.33 ± 0.34	**<0.001**	**0.004**	0.155
Leptin (ng/mL)	1.16 ± 0.14	6.49 ± 1.03 ^c^	1.27 ± 0.33	1.42 ± 0.67 ^f^	0.004	0.002	**0.003**
Adpn (μg/mL)	36.8 ± 1.9	39.9 ± 2.6	34.9 ± 4.3	36.8 ± 7.1	0.164	0.788	0.820

BW, body weight; BAT, brown adipose tissue; GWAT, gonadal white adipose tissue; HFD, high-fat diet; FER, food efficiency ratio; FI, food intake, ND, normal diet; PRWAT, perirenal white adipose tissue; SCWAT, subcutaneous white adipose tissue. Data are presented as the mean ± SEM of 6–8 animals per group. Statistical differences were analyzed by two-way ANOVA or one-way ANOVA followed by Bonferroni post hoc test if an interaction was detected. ^b^ *p* < 0.01; ^c^ *p* < 0.001 *vs*. same sex fed a ND; ^d^, *p* < 0.05; ^e^ *p* < 0.01; ^f^ *p* < 0.001 *vs*. male mice fed the same diet. * interaction between both factors (Sex and Diet). Bold lettering indicates statistically significant values.

**Table 2:** Effect of sex and diet on glucose metabolism in 12-and 32-week-old mice.

	12 WEEKS						
	Males		Females		ANOVA 12 weeks		
	ND	HFD	ND	HFD	*Sex*	*Diet*	Sex*Diet
Glucose (mg/dL)	75.2 ± 3.4	81.8 ± 4.7	70.3 ± 3.4	72.4 ± 1.6	**0.044**	0.212	0.514
Insulin (ng/mL)	0.39 ± 0.04	0.60 ± 0.05 ^b^	0.45 ± 0.05	0.39 ± 0.02 ^e^	0.104	0.106	**0.004**
HOMA-IR	0.09 ± 0.01	0.14 ± 0.02 ^a^	0.09 ± 0.01	0.08 ± 0.01 ^d^	0.038	0.140	**0.019**
QUICKI	0.67 ± 0.03	0.58 ± 0.02	0.64 ± 0.01	0.66 ± 0.01	0.282	0.202	0.034
FFA (mmol/L)	0.70 ± 0.06	0.78 ± 0.05	0.83 ± 0.14	0.85 ± 0.14	0.188	0.483	0.704
Adipo-IR	0.08 ± 0.01	0.13 ± 0.01 ^a^	0.11 ± 0.04	0.09 ± 0.01 ^c^	0.748	0.589	**0.039**

	**32 WEEKS**						
	**Males**		**Females**		**ANOVA 32 weeks**		
	**ND**	**HFD**	**ND**	**HFD**	***Sex***	***Diet***	Sex*Diet
Glucose (mg/dL)	73.0 ± 3.6	86.7 ± 4.4	65.6 ± 3.9	76.4 ± 2.1	**0.007**	**<0.001**	0.270
Insulin (ng/mL)	0.97 ± 0.13	0.86 ± 0.13	0.50 ± 0.03	0.48 ± 0.02	**0.003**	0.612	0.719
HOMA-IR	0.20 ± 0.04	0.23 ± 0.05	0.09 ± 0.01	0.11 ± 0.01	**0.014**	0.627	0.843
QUICKI	0.54 ± 0.02	0.53 ± 0.02	0.64 ± 0.02	0.61 ± 0.01	**<0.001**	0.685	0.358
FFA (mmol/L)	0.72 ± 0.06	0.75 ± 0.06	0.91 ± 0.10	0.84 ± 0.07	0.054	0.807	0.521
Adipo-IR	0.20 ± 0.03	0.21 ± 0.05	0.12 ± 0.02	0.12 ± 0.01	**0.042**	0.864	0.938

Adipo-IR, adipocyte insulin resistance index; Adpn, adiponectin; HFD, high-fat diet; HOMA, homeostasis model assessment; ND, normal diet; QUICKI, quantitative insulin sensitivity check index. Data are presented as the mean ± SEM of 6–12 animals per group. Statistical differences were analyzed by two-way ANOVA or one-way ANOVA. ^a^ *p* < 0.05; ^b^ *p* < 0.01; ^c^ *p* < 0.001 *vs*. same sex fed a ND; ^d^, *p* < 0.05; ^e^ *p* < 0.01 *vs*. male mice fed the same diet. * interaction between both factors (Sex and Diet). Bold lettering indicates statistically significant values.

## Data Availability

The data presented in this study are available upon reasonable request from the corresponding author. The data are not publicly available due to privacy restrictions.

## Supplementary Materials

The following supporting information can be downloaded at: https://www.mdpi.com/article/10.3390/nu15010073/s1. Figure S1: Experimental design.
