# Supplementary figures and images for: Sex- and Age-Dependent Changes in the Adiponectin/Leptin Ratio in Experimental Diet-Induced Obesity in Mice
Source: Nutrients. 2022 Dec 23;15(1):73. doi: 10.3390/nu15010073 (PMC9823624; doi:10.3390/nu15010073)

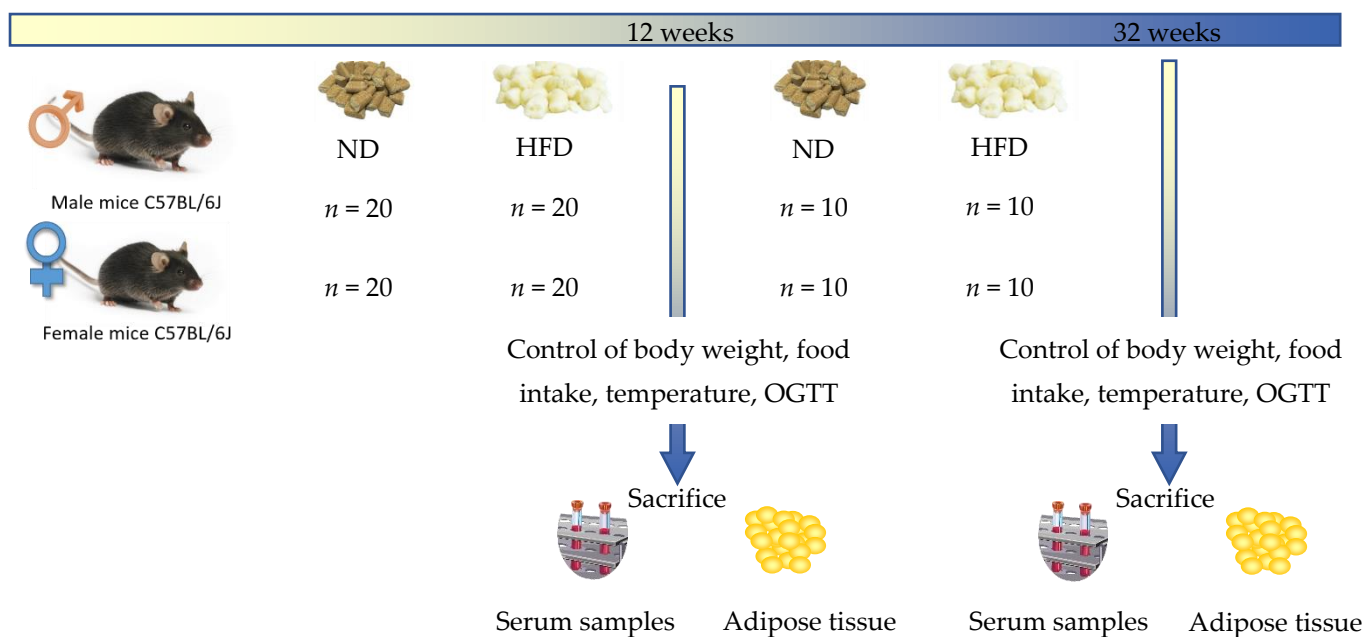

**Supplementary Figure S1.** Experimental design.

Supplement: Supplementary file 1 [file nutrients-15-00073-s001.zip › nutrients-2059075-supplementary.pdf]
